# Error inconsistency does not generally inhibit saccadic adaptation: Support for linear models of multi‐gainfield adaptation

**DOI:** 10.14814/phy2.15180

**Published:** 2022-02-25

**Authors:** Thomas Eggert, Katharina Kaltenbach, Andreas Straube

**Affiliations:** ^1^ Department of Neurology University Hospital LMU Munich Planegg/Martinsried Germany; ^2^ Department of Neurology and German Center for Vertigo and Balance Disorders‐DSGZ University Hospital LMU Munich Munich Germany

**Keywords:** adaptation, generalization, human, motor control, saccade

## Abstract

This study examined saccade adaptation induced by intrasaccadic target steps (ITS). By manipulating the ITS, we investigated potential effects of the consistency of the feedback error on saccade adaptation, which would provide evidence against the linearity of standard models of visuomotor adaptation. Previous studies addressing saccade adaptation arrived at different interpretations, but in these experiments only a single saccade amplitude was trained rather than a variety of saccade amplitudes in random order (mixed training). We extend previous studies by testing for effects of error consistency under additional control conditions described by the factors *training protocol* (single‐amplitude/mixed), *ITS direction* (onward/backward), and *adaptation phase* (training/washout). Adaptation dynamics were assessed using a model of “multi‐gainfield adaptation” developed by tailoring an existing linear model for visuomotor adaptation of movements with multiple target positions to gain adaptation of saccades with multiple amplitudes. The total adaptive change did not depend on the consistency of the ITS in either mixed or single‐amplitude training. The initial adaptation speed was lower with inconsistent ITS. However, the effect on adaptation speed occurred only during amplitude reduction and not during enlargement or washout. These results corroborate the linearity of saccade adaptation in that the mean error is the main factor determining the total adaptive change, independent of error consistency. The multi‐gainfield adaptation model was confirmed in that the retention rate and error sensitivity did not depend on the training protocol. The absence of effects of error consistency on saccade adaptation is relevant in the context of adaptive deficits in movement disorders.

## Graphical abstract

This study investigates the effects of feedback noise on saccade adaptation, a topic that is important for nonlinear extensions of standard adaptation models and addressed in previous studies, some of which arrived at different interpretations. Our study provides new insights into these differences and shows the extent to which linear models can explain saccade adaptation and its generalization across different amplitudes.

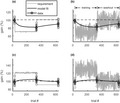

**New & Noteworthy**

- Total changes of saccade adaptation did not depend on whether the amplitudes of the intrasaccadic target steps (ITS) corresponded to a fixed or trial‐to‐trial varying percentage of the primary target steps.
- Inconsistent ITS impaired only the initial adaptation speed and only during backward training and not during onward training or during washout.
- Inspired by previous models, we developed a linear “multi‐gainfield” adaptation model to explain the invariance of the total adaptive change against ITS inconsistency.

## 1 INTRODUCTION

The adaptation of saccadic eye movements has been widely studied in the past. Saccades, i.e., rapid eye movements to fixate new points of interest, are among the most common movements we perform and are a prime example of motor adaptation because they are continuously adjusted to maintain accuracy. When an error occurs systematically, subjects adjust their eye movements progressively during the first 50–200 trials to compensate for this error. This was first demonstrated by McLaughlin (1967), who induced an artificial postsaccadic error by shifting the target intrasaccadically. The resulting visuomotor adaptation is seen as a modification of the transformation of the presaccadic visual error into the saccadic motor command (Albano, 1996; Deubel et al., 1987; Noto & Robinson, 2001; Tian et al., 2009; Wallman & Fuchs, 1998). The important role of the cerebellum in saccade adaptation has been shown by lesion studies in monkeys and in humans with cerebellar diseases (Prsa & Thier, 2011).

Standard models of visuomotor adaptation (Ethier et al., 2008a; Smith et al., 2006) assume that the incremental adaptive change depends linearly on the size of the sensorimotor feedback error. More specifically, an adaptive internal state is updated after each movement by a weighted sum of the previous state value and the sensorimotor feedback error. The weighting factor for the previous state value is called the “retention rate” and that of the feedback error is called the “error sensitivity.” Because of the linearity of such a system, the expected value of its output is identical to the system's response to the expected value of its input. Consequently, in a linear adaptation model, the expected value of the adaptive change, i.e. the output, does not depend on whether the feedback error, i.e. the input, is contaminated by mean‐free noise.

In contrast, it has also been suggested that inconsistent or noisy error signals affect adaptation in a nonlinear manner, possibly due to an assignment strategy to distinguish between internally and externally caused errors (Berniker & Kording, 2011; Herzfeld et al., 2014). A rationale behind these non‐linear adaptation dynamics is that postmovement errors that are less determined by the motor command are likely to have causes other than sensorimotor miscalibration. Therefore, it might be a useful strategy of the adaptive mechanism to reduce error sensitivity on such errors. Therefore, these non‐linear extensions of linear adaptation models predict that error inconsistency should decrease error sensitivity and thereby inhibit motor adaptation. The studies mentioned suggest that such a mechanism is a characteristic feature of sensorimotor adaptation in healthy subjects. The inhibitory effect is likely to occur only for error variabilities that are markedly larger than those that normally occur due to variability in saccade gain in healthy subjects. Therefore, such a mechanism is quite compatible with the observation that the inter‐trial dispersion of baseline saccades in healthy subjects does not correlate with the magnitude of adaptive changes (Rahmouni & Madelain, 2019). The question of whether increased error variability generally inhibits saccade adaptation in healthy individuals is also important for interpreting adaptation deficits in patients with motor disorders. For example, cerebellar degeneration diseases induce both adaptation deficits and increased motor variability (Golla et al., 2008; Straube et al., 2001; Xu‐Wilson et al., 2009). If error inconsistency resulting from motor variability inhibits adaptation because of a mechanism which is operational in patients and controls, adaptation deficits in cerebellar patients could partially be explained as a side effect of the increased motor variability. With this additional motivation in mind, in the current study we investigated whether decreased error consistency impairs saccade adaptation in healthy subjects. In different sessions, we therefore introduced different intrasaccadic target steps (ITS) with amplitudes that were either variable or a constant fraction of the primary target step.

This research question was also motivated by two previous studies on the role of error consistency in saccade adaptation, which arrived at different interpretations. Srimal et al. (2008) applied only −16 deg leftward primary target steps followed by either systematic backward (i.e., rightward) ITS of 2 deg or, in a different session, by backward (2 deg) and onward (−1 deg) ITS in random order. Under both conditions, the saccade amplitude changed with an error sensitivity (*b*) of about 0.025. Srimal et al. (2008) concluded that adaptation “is of an obligatory nature” and that it occurs even under unpredictable and random displacement of the visual target. This result suggests that error inconsistency does not inhibit saccade adaptation. In the second study, Havermann and Lappe (2010) applied +15 deg (rightward) primary target steps and directly controlled the postsaccadic retinal error that was either constant (−3, 0, or +3 deg) or variable with a gaussian distribution around the same means and two different standard deviations (2, 4 deg). This study found decreasing adaptive change with increasing standard deviation of the postsaccadic error and concluded that saccadic adaptation accounts for error consistency.

To provide a training which is as close as possible to the execution of visually guided saccades under natural viewing conditions, the training of the current study was not constrained to target steps with a single fixed start position, direction, and amplitude (*single*‐*amplitude training*), as was done in two above‐mentioned studies (Havermann & Lappe, 2010; Srimal et al., 2008). Under such constrained conditions, adaptation can be achieved by an automatized internally generated motor command that does not necessarily involve the adaptation of the visuomotor transformation. Thus, it seems desirable to avoid these constraints. Extending previous studies, we therefore applied a training protocol in which saccades with both directions and various amplitudes were performed in random order (*mixed training*). Differently from the study by Rolfs et al. (2010), we restricted all movements to the horizontal direction. In addition, the same number of saccades was used for training as for a subsequent washout. In separate sessions, different directions of ITS (onward/backward) were applied to enlarge and reduce saccade amplitude. To analyze data from such a mixed training protocol, the current study modifies an existing model for multitarget training in limb movements (Tanaka et al., 2012) for use in saccade adaptation with mixed training protocols. To compare potential effects of error consistency between the current and previous studies, we also conducted a control experiment under the conditions of a *single*‐*amplitude training*. The overall goal of the study was to investigate the influence of four different factors on saccade adaptation, namely error consistency (consistent/inconsistent), training protocol (single‐amplitude training/mixed training), ITS direction (onward/backward), and adaptation phase (training/washout). The first of these factors (consistency) was of primary interest to the study, while the other three factors served to extend previous studies by providing a variety of control conditions. The linearity of the considered models predicts that none of these factors should affect the adaptation dynamics. Conversely, any violation of this prediction indicates a non‐linearity of the adaptation mechanism.

Finally, the current study also addresses potential age effects on saccade adaptation. It has been previously observed (Huang et al., 2017) that adaptive reduction of saccade amplitude is relatively well preserved in elderly subjects. Extending this study, we also analyzed the age dependence of saccade adaptation specifically for amplitude reduction and enlargement. The widely accepted view that these two rely on different mechanisms (Collins et al., 2008; Ethier et al., 2008b; Frens & Van Opstal, 1997; Semmlow et al., 1989; Straube & Deubel, 1995) would be further supported by differences in their age dependence.

In the following, we describe (i) why we need the model extension of Tanaka et al. (2012) and its most important features, (ii) why the standard model and its extension have to be tailored for use in saccade adaptation, and (iii) how the model will be used to interpret the empirical results of this study.

There are different approaches to model adaptation in a mixed training. Both the adaptation of a single global visuomotor transformation (Cassanello et al., 2019; Rolfs et al., 2010) and parallel adaptation of several independent amplitude‐ and direction‐specific visuomotor transformations (Tanaka et al., 2012) were considered. We chose here the second approach because it allows the well‐known feature of saccade adaptation to generalize only partially to non‐trained saccade amplitudes (Noto et al., 1999; Semmlow et al., 1989; Straube et al., 1997) to be explained. Tanaka et al. (2012) developed a quantitative linear extension of the Smith‐Shadmehr model (Smith et al., 2006) for adaptation of limb movements to multiple targets which requires careful consideration of the exact frequency and the order of the amplitudes of the trained saccades and is characterized by the following features: (1) The adaptive internal memory state of the standard model is replaced by a set of internal states, each specific to a range of target positions. (2) The updating of each internal state follows the same rule as the standard linear adaptation model, with both retention rate and error sensitivity being the same for all states. (3) Every post‐movement error contributes to the adaptation of all internal states with a linear weighting that decreases with increasing distance between the amplitude of the current movement and the center of the amplitude range of the respective state. Because this model extension preserves the linearity of the standard model (Smith et al., 2006), it also preserves its invariance to contamination of the sensorimotor feedback by noise. To our knowledge, the model extension (Tanaka et al., 2012) has not been used extensively in saccade adaptation.

A possible reason for this is that saccade adaptation is traditionally viewed as a gain adaptation with limited generalization to adjacent amplitudes, characterized by the so‐called “gain adaptation field” (Noto et al., 1999). In contrast, both the Smith‐Shadmehr model (Smith et al., 2006) and its extension by Tanaka et al. (2012) were formulated as amplitude‐adaptation models. The main difference between these two model‐subtypes is the internal memory variable which represents the adaptive change of either saccade amplitude or saccade gain. Differences between amplitude adaptation and gain adaptation will be treated in more detail in the Section 4. The modeling section in Section 2 describes the modification of the multi‐target adaptation model (Tanaka et al., 2012) for use in gain adaptation. The resulting model is referred to here as the “multi‐gainfield adaptation model” because each component of its state vector represents the gain of all saccades within a given amplitude range, i.e., within the respective “gainfield.” In addition, the modelling section will also analyze predictions of the multi‐gainfield adaptation model for different training protocols (mixed training or single amplitude training). Thereby, it is important to note that even protocols with single‐amplitude training require models of multi‐gainfield adaptation because they always involve reset saccades with a different direction than the trained saccades. Therefore, the multi‐gainfield adaptation model is required to account correctly for the retention loss that occurs on the trained gain field during the reset saccades.

The use of this multi‐gainfield adaptation model in the current study has several purposes: First, it is used to parameterize the experimentally observed adaptation by estimating the total amount and the time constant of the adaptive change in our mixed training protocol. This parameterization allows the role of error consistency in saccade adaptation to be investigated. Insensitivity of the fitted parameters to differences in error consistency can be interpreted as confirmation of the linearity of the standard adaptation model (Smith et al., 2006). Second, during mixed training, the prediction of the multi‐gainfield model concerning the adaptation dynamics of different saccade amplitudes will be compared with the empirical data. Agreement between both is interpreted as support for the model. Third, the multi‐gainfield adaptation model will be further tested by comparing the estimates of error sensitivity and retention rate between the mixed training and the single‐amplitude training. Invariance of these model parameters against the training protocol is interpreted as support for the model.

## 2 METHODS

### 2.1 Subjects

A total of 16 subjects participated in this study (9 women, 7 men; ocular dominance: 12 right, 4 left). None of the subjects had a known visual, neurological, or psychiatric disorder. Their vision was either normal or corrected to normal and was tested with a Snellen test. Ocular dominance was assessed by the “hole‐in‐the‐card test” (Li et al., 2010). Each subject belonged to one of two age groups (22–35 years, *N* = 9; 53–64 years, *N* = 7). All subjects except two were naïve to saccade studies, gave informed consent, and were compensated for the participation in all 4 sessions. A subset of 5 subjects (age: 24–64 years; 3 naïve) also participated in a second experiment, thus providing comparable data for both experiments. Subjects gave written informed consent to the study that complied with the Declaration of Helsinki and was approved by the ethics committee of the medical faculty of the Ludwig‐Maximilians Universität (project number: 20‐576).

### 2.2 Setup

The subjects were seated at a distance of 160 cm from a projection screen. The visual target spot (diameter 0.1 deg) projected onto this screen was generated by a red laser diode and controlled by a mirror galvanometer (General Scanning G120D, Watertown, MA, USA), which can execute a step of 20 deg amplitude in less than 2 ms (absolute position error < 0.04 deg). The scanner signal was generated by a real time control system *REX* (Hays et al., 1982) for analog and digital input/output running at a frequency of 1 kHz. Horizontal eye movements were recorded by a camera system at a frequency of 305 Hz (Dera et al., 2006) and were mapped and upsampled to the recording frequency of 1 kHz by linear interpolation. The viewing was binocular and both eyes were recorded. For all subjects, the dominant eye was analyzed. To avoid any visual references the room was dark during the entire session. The head was stabilized on a chin rest.

### 2.3 Task and paradigm

**FIGURE 1:**
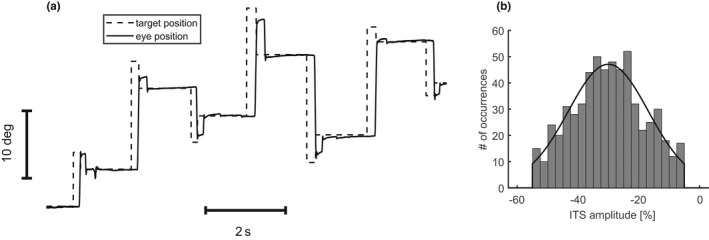
Illustration of the mixed training paradigm with backward and inconsistent intrasaccadic target steps (ITS). Four primary target amplitudes (±8, ±16 deg) were presented in randomized order. Each ITS amplitude, expressed as a percentage of the respective primary step amplitude, was drawn from the random distribution shown in (b). Solid: horizontal eye position. Dash‐dotted: horizontal target position

In a modified McLaughlin paradigm (McLaughlin, 1967), the subjects attempted to follow a fixation target that stepped to various positions on the horizontal meridian. During the subject's saccade toward the peripheral target, it was horizontally displaced to induce an artificial postsaccadic error. The secondary target position of each trial served as the fixation position of the next trial. The principle of this paradigm is illustrated in Figure 1.

Subjects were instructed to fixate and follow the target as precisely and fast as possible, maintaining fixation as long as possible until the occurrence of next target step. Subjects exhibiting anticipatory saccades were admonished to refrain from doing so. The primary target step was executed with a random delay between 950 and 1050 ms after the previous primary saccade, which was detected online. The ITS, if provided, was released by an online trigger generated by the real‐time control system *REX* at the time when the eye velocity dropped below 90% of the peak velocity. Since the availability of the target immediately after the saccade is crucial for the visibility of the secondary target step (Deubel et al., 1996, 1998) as well as for the generation of corrective saccades (Eggert et al., 1999), it is very important to ensure that the ITS terminates before the end of the primary saccade. Our trigger mechanism fulfilled this condition as shown by the following analysis: The online computation of the eye velocity was done with a five‐point differentiator which caused the eye‐velocity signal to be delayed by 2 frames of the video‐eyetracker (2 × 1/305 = 7 ms). Taking into account the additional delay of the video eye tracker due to the exposure time of the CCD‐sensor, the processing time of the pupil detection algorithm (5 ms), and the loop time (1 ms) of the REX real‐time control system, we were able to ensure that the secondary target position was reached in less than 15 ms after the peak velocity and thus well before the end of the primary saccade. Offline analysis confirmed that even for the small primary target steps of 8 deg, the secondary target step was completed approximately 5 ms before saccade termination when the eye velocity was still larger than 50 deg/s.

Each session started with 30 normal baseline trials without ITS. In the following 300 adaptation trials, we introduced an ITS in the direction (onward) of or opposite (backward) to the primary target step. After this training, there were another 300 trials without intrasaccadic target steps, further referred to as “washout.” Each subject performed four such adaptation sessions which differed with respect to the direction of the ITS during the training (onward/backward) and with respect to the consistency of the ITS (consistent/inconsistent). To minimize possible order‐induced biases, we used a Latin square design to determine four possible sequences in which each of the conditions appeared exactly once at each of the four sequence positions. Each of the 16 participants was assigned to a sequence so that each of the four sequences was applied in four participants. The different sessions were each separated by approximately seven days.

### 2.4 Consistency of the intrasaccadic target step

The main interest of our study was the dependence of adaptation on the consistency of the postsaccadic error, which was manipulated in our experiment by varying the ITS. In four different sessions, both onward and backward ITS were examined, each in a condition with consistent ITS and another one with inconsistent ITS. The amplitude of the consistent ITS was 30 ± 0% of the amplitude of the primary target step. In the inconsistent ITS‐condition, the ITS‐amplitudes were randomly distributed between 5% and 55% of the primary target amplitude with a mean and standard deviation of 30 ± 11%. In both conditions, the mean ITS was identical (30%). For target amplitudes of 16 deg, the median [interquartile range] of the standard deviations of the resulting postsaccadic errors was 1.0 [0.37] deg and 2.1 [0.22] deg for the conditions with consistent and inconsistent ITS, respectively.

### 2.5 Experiments

We conducted two experiments, both consisting of the four sessions described above, but differing in the distributions of target positions and amplitudes of primary target steps and in the distributions of ITS.

In Experiment 1, a mixed training was applied. The target steps had an amplitude of either ±8 or ±16 deg, with negative amplitudes indicating a movement to the left and positive ones to the right. The number of target steps with absolute amplitudes of 8 deg was equal to those with 16 deg and presented in randomized order. The direction of the target step was also randomized with the constraint that the target position remained within a range of ±25 deg. Each primary saccade was accompanied by an ITS.

Five of the 16 subjects also participated in Experiment 2, in which a single‐amplitude training was applied. Every second target step had a purely horizontal rightward amplitude of 16 deg and started at the vertical meridian (horizontal position 0 deg). Only these target steps were accompanied by a secondary ITS, whereas the target steps returning to the vertical meridian were not. To induce some variability in primary target positions, the vertical eccentricity of the target in its starting position on the vertical meridian was varied between ±5 deg. Thus, in contrast to the purely horizontal centrifugal saccades in Experiment 1, the non‐adapted return saccades had a small vertical component. Experiment 2 was designed as a within‐subject control that was conducted after the completion of Experiment 1. Therefore, all five subjects who participated in the second experiment performed this control after Experiment 1.

### 2.6 Modeling and data analysis

We fitted the adaptation time course with the standard single‐state model of Smith et al. (2006), applied to the saccade gain rather than to saccade amplitude. The saccade gain (g) was defined as the ratio between the saccade amplitude (A) and the amplitude (R) required to accurately hit the target at its primary position. By using this procedure, we followed the traditional notion of a gain adaptation (Deubel et al., 1986, 1987; Kojima et al., 2004). The main parameters of the Smith‐Shadmehr model are the error‐sensitivity ($b_{s}$), the retention rate ($a_{s}$), the variance ($w_{\mathrm{ss}}^{2}$) of the planning noise ($w_{n}$), and the variance ($\sigma^{2}$) of the execution noise $v_{n}$. The application of the model in gain‐adaptation is described in the following.

#### 2.6.1 The single‐state adaptation model

Applying the single‐state model to the gain implies that both the adaptive change (x) of the saccade amplitude (A) and the error signal (e) driving the adaptation are expressed as a fraction of the required amplitude (R). In that model, the adaptive gain change (x) is driven by the normalized postsaccadic retinal error ($\varepsilon =\frac{e}{R}$). Thereby, both the adaptive change x and the normalized retinal error ε are expressed as the differences from the baseline values prior to adaptation. The update of the gain change from trial *n* to the next trial (*n* + 1) is defined as the sum of the retention of the fraction $a_{s}$ of the previous gain change $x\left(n\right)$ and the normalized error $\varepsilon \left(n\right)$, weighted by the error sensitivity $b_{s}$. This update is contaminated by the planning noise $w\left(n\right)$:

(1a) $$x\left(n+1\right)=a_{s}x\left(n\right)+b_{s}\varepsilon \left(n\right)+w\left(n\right)$$

The output of the model is the saccade gain $g\left(n\right)$ which is, for each trial *n*, defined as the sum of the baseline gain $g_{b}$ prior to any adaptation and the adaptive gain change $x\left(n\right)$. This process is contaminated by execution noise $v\left(n\right)$:

(1b) $$g\left(n\right)=g_{b}+x\left(n\right)+v\left(n\right)$$

Both the planning noise $w\left(n\right)$ and the execution noise $v\left(n\right)$ were expressed in terms of the gain (dimensionless units) and were assumed to be normally distributed:

(1c) $$w\left(n\right)∼N\left(0,w_{\mathrm{ss}}^{2}\right)\;v\left(n\right)∼N\left(0,\sigma^{2}\right)$$

with constant variances $w_{\mathrm{ss}}^{2}$ and $\sigma^{2}$. The saccade amplitude (A) is the product of the gain with the required amplitude

(1d) $$A\left(n\right)=g\left(n\right)R\left(n\right)$$

and the normalized error is

(1e) $$\begin{array}{cc} \varepsilon \left(n\right) & =\frac{e\left(n\right)}{R\left(n\right)}=\frac{g_{b}R\left(n\right)+ITS\left(n\right)‐A\left(n\right)}{R\left(n\right)}\\ & =g_{b}+\frac{ITS\left(n\right)}{R\left(n\right)}‐g\left(n\right)=u\left(n\right)‐x\left(n\right)‐v\left(n\right) \end{array}$$

Here, $u\left(n\right)=\frac{ITS\left(n\right)}{R\left(n\right)}$ denotes the normalized ITS. The gain requirement is defined as that saccade gain for which the normalized error vanishes ($g_{b}+u\left(n\right)$). Equation (1e) shows that the adaptation is driven by the difference between the driving external stimulus $u\left(n\right)$ and the adaptive gain change $x\left(n\right)$, contaminated by the execution noise $v\left(n\right)$. Equation (1) models the dynamics of the adaptive gain change $x\left(n\right)$ by a discrete‐time linear filter which can be summarized by

(2a) $$x\left(n+1\right)=\left(a_{s}‐b_{s}\right)x\left(n\right)+b_{s}\left(u\left(n\right)‐v\left(n\right)\right)+w\left(n\right)$$

(2b) $$g\left(n\right)=g_{b}+x\left(n\right)+v\left(n\right)$$

Note that the mechanism of gain adaptation, as expressed in Equations (1) and (2), results from the standard model of amplitude adaptation (Smith et al., 2006) by dividing the state variable x and the driving signal u but not the noise components (v and w) by the required amplitude (R). Thus, the gain adaptation formulated in Equations (1) and (2) can equivalently be expressed by an amplitude adaptation with noise components whose standard deviations are not constant (see Equation (1c)), but proportional to the required amplitude. In a previous study (Eggert et al., 2016), we argued that planning noise of saccades is signal‐dependent in that way because the local magnification factor of the primary visual cortex and sensory motor maps such as the superior colliculus increases linearly with target eccentricity. From this point of view, the described mechanism of gain adaptation with constant noise is an equivalent of amplitude adaptation with signal proportional noise. But beyond that, gain adaptation makes additional predictions about how adaptive changes generalize to adjacent non‐trained amplitudes.

For a constant normalized ITS (u), Equation (2) predicts an exponential time course of the expected gain

(3) $$E\left\{g\left(n\right)\right\}=g_{b}+x\left(0\right)\;\exp\left(‐\frac{n}{\tau }\right)+uG\left(1‐exp\left(‐\frac{n}{\tau }\right)\right)$$

with the time constant

(4a) $$\tau =‐log^{‐1}\left(a_{s}‐b_{s}\right)$$

expressed in the units of trial number, and the adaptation gain (G)

(4b) $$G=\frac{b_{s}}{1‐a_{s}+b_{s}}$$

In general, for time‐varying normalized ITS, the expected gain $E\left\{g\left(n\right)\right\}$ can be calculated by iteratively evaluating Equation (2), while setting the noise signals $w\left(n\right)$ and $v\left(n\right)$ to zero. The total gain change was defined as

(5) $$\Delta g\left(n\right)=E\left\{g\left(n\right)‐g\left(0\right)\right\}=E\left\{x\left(n\right)‐x\left(0\right)\right\}$$

This single‐state model is the basis for understanding the role of error sensitivity and retention rate in saccade gain adaptation, but it is not sufficient to describe saccadic adaptation in more complex training protocols involving multiple gain fields.

#### 2.6.2 Multi‐gainfield adaptation

In Experiment 1, saccades of different directions and amplitudes were performed in random order, whereas in Experiment 2 saccades of ±16 deg alternated regularly. It is well known that saccadic adaptation is specific to amplitude and direction and that it generalizes to non‐trained saccades only within limited “gain adaptation fields” (Deubel et al., 1987; Noto et al., 1999; Straube et al., 1997). Since this effect is essential for comparing adaptation between the training protocols of Exp. 1 and Exp. 2, we modeled adaptation under the consideration of four adaptive gain fields associated with the four primary target step amplitudes [−16, −8, +8, +16] deg. The adaptive changes of these gain fields were represented by the components of the four‐dimensional state vector ${\underline{x}}_{n}$, where the dynamics of each of these states was determined by the same retention rate ($a_{s}$) and the same error sensitivity ($b_{s}$). Thereby, we followed the approach of Tanaka et al. (2012) for modeling multi‐target adaptation. In this model, each postsaccadic error contributes to the adaptation of all gain fields with a linear weighting factor which decreases with increasing distance of saccade amplitude from the center of the gain field. The model does not consider any non‐linear interaction (inhibition or excitation) between adjacent gain fields. A characteristic feature of this model is that error processing and adaptation dynamics are considered as separable mechanisms. This is reflected in the fact that the error weights (C) and the parameters of the adaptation dynamics ($a_{s}$, $b_{s}$) form two separate sets of parameters. Consequently, for a given set of weights, the remaining parameters to be estimated are still the same as in the basic model of the previous section ($a_{s}$, $b_{s}$, $\sigma^{2}$, $w_{\mathrm{ss}}^{2}$). Applying this approach in “multi‐gainfield adaptation,” Equation (2) extends to a vector equation.

(6a) $$\underline{x}\left(n+1\right)=A\left(n\right)\underline{x}\left(n\right)+\underline{b}\left(n\right)\left(u\left(n\right)‐v\left(n\right)\right)+\underline{w}\left(n\right)$$

(6b) $$g\left(n\right)={\underline{\delta }}^{T}\left(n\right)\left[{\underline{g}}_{b}+\underline{x}\left(n\right)\right]+v\left(n\right)$$

with the time variant coefficients $A\left(n\right)$ (dimension: 4 × 4), $\underline{b}\left(n\right)$ (dimension 4 × 1), and $\underline{\delta }\left(n\right)$ (dimension 4 × 1). The 4 × 1 vector ${\underline{g}}_{b}$ contains the baseline values of the four gain fields and the vector $\underline{\delta }\left(n\right)$ indicates the class membership of the external adaptation stimulus: The k‐th component of $\underline{\delta }\left(n\right)$ equals one and all other components equal zero when the target amplitude in trial *n* belongs to the class *k*. The coefficients $A\left(n\right)$ and $\underline{b}\left(n\right)$ were defined as follows:

(7a) $$\underline{b}\left(n\right)=b_{s}C\underline{\delta }\left(n\right)$$

(7b) $$A\left(n\right)=a_{s}I‐\underline{b}\left(n\right){\underline{\delta }}^{T}\left(n\right)$$

where the matrix $C=\left[c_{\mathrm{ik}}\right]$ denotes the relative transfer of the error $\varepsilon \left(n\right)$, evoked by a target amplitude of class *k* to the adaptation of the gain field *i* ($0\leq c_{\mathrm{ik}}\leq 1$). Based on the results of Straube et al. (1997) we used the following transfer matrix for the target amplitudes [−16, −8, +8, +16] deg:

(8) $$C=\left[\begin{array}{cccc} 1.00 & 0.40 & 0 & 0\\ 0.53 & 1.00 & 0 & 0\\ 0 & 0 & 1.00 & 0.53\\ 0 & 0 & 0.40 & 1.00 \end{array}\right]$$

The likelihood that the model (Equations (6)–(8)) generated the observed gain sequence $g\left(n\right)$ (0≤n≤N) was then computed and minimized as a function of the parameters ($a_{s}$, $b_{s}$, $\sigma^{2}$, $w_{\mathrm{ss}}^{2}$). This model fit of the time variant system (Equations (6)–(8)) was performed with a maximum‐likelihood approach (Eggert et al., 2021) which allows estimating not only the error‐sensitivity ($b_{s}$) and the retention rate ($a_{s}$), but also the variance ($w_{\mathrm{ss}}^{2}$) of the planning noise and the variance ($\sigma^{2}$) of the execution noise.

The training protocol of Experiment 1 was a “mixed training” defined by the two features that (1) all gain fields were trained with equal probabilities in random order, and that (2) the expected normalized ITS ($E\left\{u\left(n\right)\right\}$) was constant. For such a “mixed training,” the multi‐gainfield adaptation model predicts that the expected time course of the saccade gain $E\left\{g\left(n\right)\right\}$ is strictly exponential only if the transfer matrix C is symmetric, and if the initial values of all gain fields are all identical to each other. In that case, all expected gain fields stay identical across the entire training period and follow an exponential time course as in Equation (3). The time constant of this exponential is:

(9a) $$\tau_{\bar{g}}=‐log^{‐1}\left(a_{s}‐{\bar{b}}_{s}\right)$$

and the adaptation gain is

(9b) $$G_{\bar{g}}=\frac{{\bar{b}}_{s}}{1‐a_{s}+{\bar{b}}_{s}}$$

with

(9c) $${\bar{b}}_{s}=b_{s}\frac{1}{m^{2}}\sum_{i,=1}^{m}\sum_{k=1}^{m}c_{i,k}$$

where *m* denotes the number of gain fields (*m* = 4 in Exp. 1).

**FIGURE 2:**
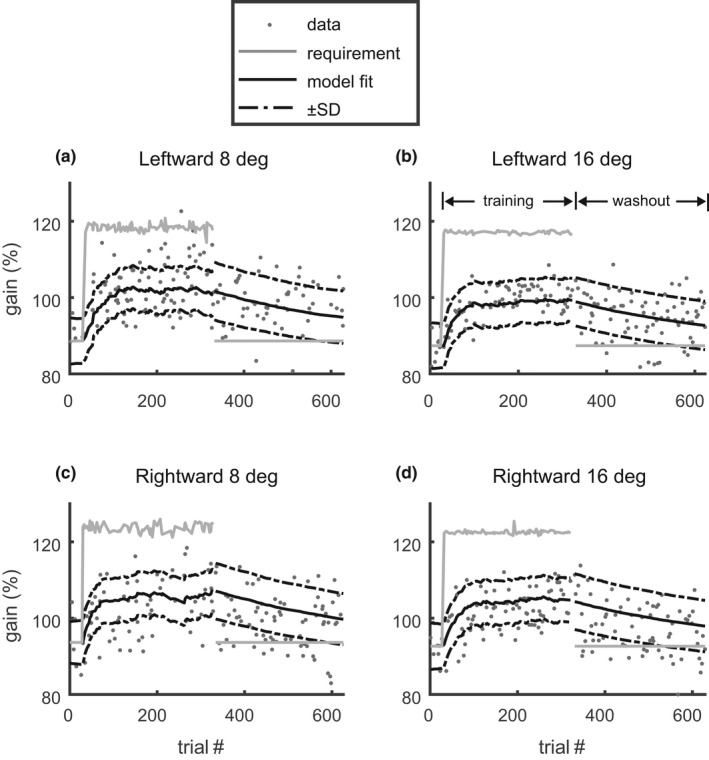
Gain adaptation in a single subject to onward, consistent ITS with an amplitude of −30% of the primary target amplitude in “mixed training” and subsequent washout (Exp. 1). The four plots show the gains in response to the four primary target step amplitudes (a: −8 deg, b: −16 deg, c: +8 deg, d: +16 deg) which were presented with equal frequency and mixed in random order. Dots: gain of individual saccades. Solid gray line: gain requirement, i.e. the respective component of ${\underline{g}}_{b}+u\left(n\right)$. The small variations in the gain requirement originate from errors of the presaccadic fixation inducing small variations in $R\left(n\right)$ and thereby also in $u\left(n\right)$. Solid black line: model prediction of the multi‐gainfield adaptation model, i.e. the four components of ${\underline{g}}_{b}+{\underline{x}}_{n}$. The four gain fields adapted in parallel and by similar amounts

These conditions were nearly met in Experiment 1, as confirmed by Figure 2, which shows that the adaptation occurred nearly in parallel in all four gain fields. This pattern was observed in onward adaptation (Figure 2) and in backward adaptation. Therefore, in Experiment 1, we quantified the adaptation dynamics by reporting the time constant $\tau_{\bar{g}}$. The total gain change was quantified by the average of the adaptive changes across all four gain fields:

(10) $$\Delta \bar{g}\left(n\right)=\frac{1}{4}\left[1,1,1,1\right]E\left\{\underline{x}\left(n\right)‐\underline{x}\left(0\right)\right\}$$

$\Delta \bar{g}\left(n\right)$ was calculated iteratively by evaluating Equation (6a) and Equation (10), while setting the noise signals $w\left(n\right)$ and $v\left(n\right)$ to zero.

In Experiment 2, we used a “single‐amplitude training,” defined as a training protocol in which only a single class of target steps is associated with a constant normalized ITS (u) and every second target step is rightward. In Experiment 2, we used only two gain fields, one for rightward and one for leftward saccades without cross‐transfer sensitivity in between $\left(C=\left[\begin{array}{cc} 1 & 0\\ 0 & 1 \end{array}\right]\right)$. In Experiment 2, the gain change of the trained saccades is represented by the second component of the two‐dimensional state vector $\underline{x}\left(n\right)$. Accordingly, the total gain change of the trained saccades is:

(11) $$\Delta g_{2}\left(n\right)=\left[0,1\right]E\left\{\underline{x}\left(n\right)‐\underline{x}\left(0\right)\right\}$$

With this, Equation (11) is solved to

(12a) $$\Delta g_{2}\left(n\right)=\left(uG_{2}‐x_{2}\left(0\right)\right)\left(1‐exp\left(‐\frac{n}{\tau_{2}}\right)\right)$$

with the time constant

(12b) $$\tau_{2}=‐2log^{‐1}\left(a_{s}^{2}‐a_{s}b_{s}\right)$$

expressed in the units of trial number and the adaptation gain ($G_{2}$)

(12c) $$G_{2}=\frac{a_{s}b_{s}}{1‐a_{s}^{2}+a_{s}b_{s}}$$

In general, for time‐varying normalized ITS, $\Delta g_{2}\left(n\right)$ was calculated iteratively by evaluating Equation (6a) and Equation (11), while setting the noise signals $w\left(n\right)$ and $v\left(n\right)$ to zero.

The above modeling shows that the multi‐gainfield model of Tanaka et al. (2012) predicts how $\Delta \bar{g}\left(n\right)$ and $\Delta g_{2}\left(n\right)$ differ between the training protocols of Exp. 1 and Exp. 2. Qualitatively, this effect can be evaluated by the differences between Equations ((9a), (9b), (9c)) and Equations ((12a), (12b), (12c)) for identical error sensitivity $b_{s}$ and identical retention rate $a_{s}$.

#### 2.6.3 Offline analysis and dependent variables

In the offline analysis, the start and end of each primary saccade were defined as the time when the eye velocity increased above or fell below 10% of saccade peak velocity. The saccade amplitude ($A_{n}$) was computed as the difference of the eye position between the end and the start of the saccade. The required saccade amplitude ($R_{n}$) was computed as the difference between the target position and the eye position at the start of the saccade. Trials in which the saccade occurred earlier than 80 ms after the target step were considered anticipatory and therefore excluded, as were saccades in the opposite direction to the target step and saccades lasting longer than 200 ms. Trials were also marked as “invalid” if the saccade gain exceeded the range mean ± 3·SD computed within a window of ±4 trials around the current trial. Based on these criteria, on average 14 ± 6% of the trials were not used for the estimation of the model parameters ($a_{s}$, $b_{s}$, $\sigma^{2}$, $w_{\mathrm{ss}}^{2}$). However, our estimation procedure (Eggert et al., 2021) accounts correctly for missing values. The estimation of the four parameters was performed separately for each subject, for each session, and separately for the training and for the washout adaptation phases. Trials 30–329 were fitted for the training under the constraint ${\underline{x}}^{t}\left(0\right)=\underline{0}$, and trials 330–629 were fitted for the washout under the continuity constraint that the initial gain change of the washout model (${\underline{x}}^{w}\left(0\right)$) was identical to the final gain change of the training model (${\underline{x}}^{t}\left(300\right)$). The baseline gain was the same for both training and washout and was computed from the 30 initial trials before the beginning of the training. In Experiment 1, the following dependent variables were extracted from each model fit:

1. $\left|\Delta \bar{g}\left(300\right)\right|$: the absolute total gain change in % (see Equation (10)).
2. $\tau_{\bar{g}}$: the time constant (in trials) (see Equation (9a)).

The absolute total gain changes were computed according to Equation (10) and multiplied by minus one for the training in the backward session and for the washout in the onward session. The time constants were computed using Equation (9).

The corresponding dependent variables in Experiment 2 were:

1. $\left|\Delta g_{2}\left(300\right)\right|$: the absolute total gain change in % (see Equation (11)).
2. $\tau_{2}$: the time constant (in trials) (see Equation (12b)).

The modelling section showed how, according to Tanaka et al. (2012), the adaptive gain change is expected to differ between the “mixed training” ($\Delta \bar{g}\left(n\right)$, Exp. 1) and “single‐amplitude training” ($\Delta g_{2}\left(n\right)$, Exp. 2) if the error sensitivity $b_{s}$ and the retention rate $a_{s}$ are considered as subject‐specific parameters that do not depend on the training protocols. For a single subject, the estimates of $a_{s}$ and $b_{s}$ obtained by maximizing the likelihood of $g\left(n\right)$ (Equation (6)) are therefore not expected to differ between Exp. 1, and Exp. 2. Vice versa, any within‐subject difference of $a_{s}$ and $b_{s}$ between the experiments would suggest that the interaction between the different gain fields were not correctly modeled.

### 2.7 Statistics

To analyze the effects of the direction and the consistency of the ITS on adaptation in Experiment 1, we submitted the absolute gain changes ($\left|\Delta \bar{g}\left(300\right)\right|$) and the time constants ($\tau_{\bar{g}}$) from Exp. 1 to a repeated measures ANOVA with the repeated factors *direction* (onward/backward session), *phase* (training/washout), and *consistency* (consistent/inconsistent) of the ITS.

The comparison of the adaptive systems between Exp. 1 and Exp. 2 was performed by subjecting the absolute total gain change during the training ($\left|\Delta \bar{g}\left(300\right)\right|$, respectively $\left|\Delta g_{2}\left(300\right)\right|$) to a repeated measures ANOVA with the repeated factors *direction* (onward/backward) and *experiment* (1/2). The same factorial analysis was performed on the retention rates ($a_{s}$) and error sensitivities ($b_{s}$) fitted to the adaptive gain changes in Exp. 1 and Exp. 2.

The normality of the distributions of the dependent variables was checked using the Lilliefors‐Test, which confirmed the normality of the absolute total gain changes but rejected that of the time constants and the model parameters $a_{s}$ and $b_{s}$. Therefore, the factorial analyses of the time constants and of the model parameters were performed using the aligned rank transform (ART) for a repeated measures design (Bortz et al., 1990; Wobbrock et al., 2011). Paired post‐hoc comparisons were performed with the paired *t*‐test for the normally distributed total gain changes, and with the Wilcoxon signed‐rank test for the non‐normal time constants, retention rates, and error sensitivities. Throughout the paper, the normally distributed variables are characterized by mean ± standard deviation, whereas the non‐normally distributed variables are characterized by *median* [*interquartile range*].

The dependence of the absolute total gain change ($\left|\Delta \bar{g}\left(300\right)\right|$) on age was analyzed by means of a repeated measures model including the within‐subject categorical factor *direction* (onward/backward) and the between‐subjects continuous predictor *age*. The repeated measures model was computed using the function *fitrm* of the “Statistics and Machine Learning Toolbox” (Matlab^®^, Mathworks, Natick, MA, USA). To evaluate the age dependency separately for both adaptation directions, two linear regressions of the adaptive change were computed, one for onward and one for backward adaptation.

## 3 RESULTS

### 3.1 Multi‐gainfield adaptation

**FIGURE 3:**
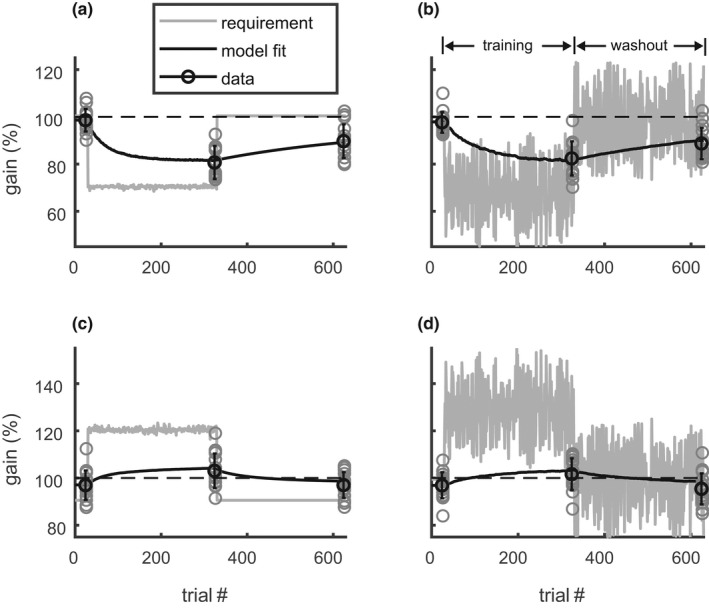
Average of the time courses of gain adaptation in the four sessions with “mixed training” and subsequent washout (Exp. 1) across all 16 subjects. (a) backward‐consistent ITS (−30 ± 0%). (b) backward‐inconsistent ITS (−30 ± 11%). (c) onward‐consistent ITS (+30 ± 0%). (d): onward‐inconsistent (+30 ± 11%). Gray circles: individual saccadic gains averaged across the last 10 trials of the baseline, training, and washout. Black circles/Whiskers: group mean ± SD. Solid black lines: expected saccade gain as predicted by the multi‐gainfield model (Equations (6a) and (6b)). The line shows the average across all four gain fields and across all subjects. Two different sets of parameters ($a_{s}$, $b_{s}$, $x_{0}$, $\sigma^{2}$, $w_{\mathrm{ss}}^{2}$) were fitted to the training (trials 30–329) and the washout (trials 330–629). Solid gray: The required gain, averaged across all gain fields: $\frac{1}{4}\left[1,1,1,1\right]\left({\underline{g}}_{b}+u\left(n\right)\right)$

Figure 3 shows the average adaptation time course across all 16 subjects, expressed as the average of the four gain fields (black solid: $\frac{1}{4}\left[1,1,1,1\right]\left({\underline{g}}_{b}+\underline{x}\left(n\right)\right)$). This plot illustrates that the subjects adapted more strongly in the gain‐decrease paradigm (Figure 3a and b, error direction backward) than in the gain‐increase paradigm (Figure 3c and d, error direction onward). This difference was observed under both conditions with consistent and with inconsistent ITS.

**FIGURE 4:**
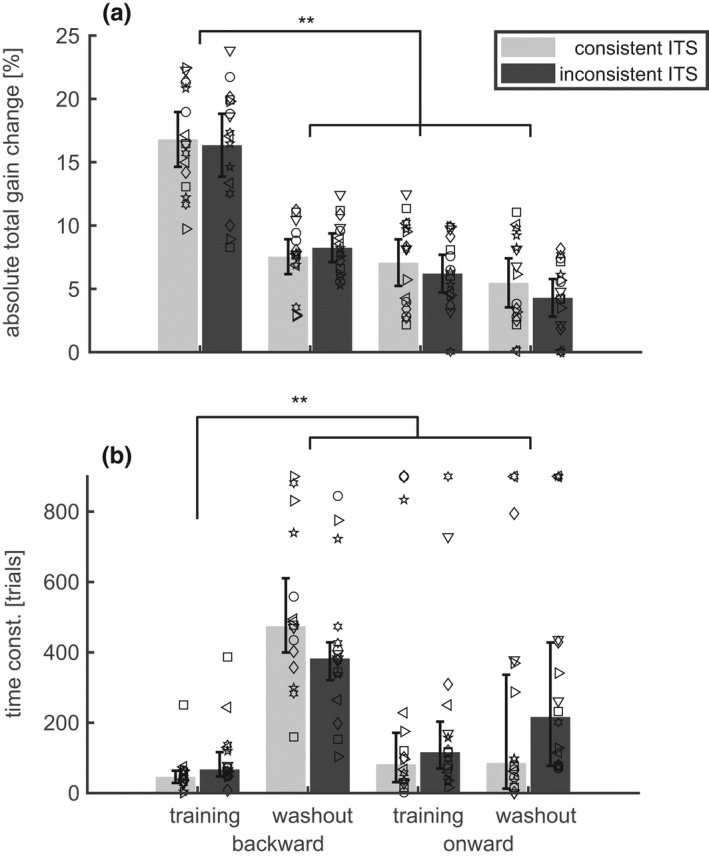
Adaptation dynamics in “mixed training” and subsequent washout (Exp. 1). (a) The absolute value of the total adaptive gain change $\left|\Delta \bar{g}\left(300\right)\right|$. Bars and whiskers show the mean and the 95% confidence range of the mean. (b) The time constant $\tau_{\bar{g}}$. Bars and whiskers show the median and the 95% confidence range of the median. Both dependent variables are shown for both directions (onward/backward) of the ITS, and for both conditions (consistent/inconsistent ITS) and for each of the 16 subjects (symbols). The total adaptive gain change was larger, and the time constant was smaller in backward training than in all other conditions. The consistency of the ITS had no significant effect on the adaptation. Asterisks indicate significant differences in the paired post‐hoc comparisons (**p < 0.01)

The population statistics of the parameters of the adaptation dynamics are shown in Figure 4. The absolute total gain change during the backward training ($\left|\Delta \bar{g}\left(300\right)\right|$) was 16.6 ± 4.1% and was almost double than the total adaptive change that occurred during the other 3 conditions (backward/washout: 7.9 ± 4.1%; onward/training: 6.6 ± 2.8%; onward/washout: 4.9 ± 2.6%). Statistically, this was reflected by the highly significant main effects of the factors *direction* (F(1,15) = 61.8; *p* < 0.0001) and *phase* (F(1,15) = 107.0; *p* < 0.0001), and the interaction between both factors (F(1,15) = 47.5; *p* < 0.0001). Thus, the absolute total gain change did not just depend on the sign of the error (ε) but also on the adaptation phase. This is particularly evident in that the absolute total gain change during the backward training (ε<0) was not only larger than the changes occurring during conditions with positive error (backward/washout or onward/training; paired *t*‐test: *p* < 0.0001) but was also larger than during the washout after onward training (paired *t*‐test: *p* < 0.001) during which the error was negative. The factor *consistency* had no significant effect on the size of the adaptive change and neither did any interaction including this factor. Because of this insensitivity of the total adaptive change to the consistency factor, the mean postsaccadic error also did not differ significantly between the conditions with consistent and inconsistent ITS.

The special role of the backward training condition was also shown by the time constant $\tau_{\bar{g}}$ (Figure 4b) whose median was smaller in this (58.05 [50.43] trials) than in all other conditions (backward/washout: 434.48 [140.05] trials; onward/training: 113.89 [361.43] trials; onward/washout: 181.87 [334.66] trials). The ART‐ANOVA applied to the time constants showed the following significant effects: A main effect of the factor *phase* (χ^2^(1) = 21.1; *p* < 0.0001), an interaction between the factors *direction* and *phase* (χ^2^(1) = 15.3; *p* < 0.0001). These effects were due to the shortening of the time constant that occurred specifically during the backward/training condition. A third order interaction between the factors *direction*, *phase*, and *consistency* (χ^2^(1) = 5.29; *p* = 0.021). This third‐order interaction was due to the non‐significant tendency of smaller time constant with inconsistent rather than with consistent ITS during washout after backward adaptation (second bar group in Figure 4b). This interaction vanished when the ART‐ANOVA was repeated without these two conditions. The effects of the factor *consistency* on the time constants were much smaller than the strong second order interaction between the *direction* and *phase* factors. Since the ART‐ANOVA on the time constant did not reveal any other significant effect or interaction involving the factor *consistency*, we conclude that main effect revealed by Experiment 1 was that backward adaptation under training showed larger and faster changes of the saccade gain than all other tested adaptation conditions. The consistency of the ITS did not affect saccade adaptation.

The ART‐ANOVA applied to the fitted error sensitivity ($b_{s}$) showed significant main effects of the factors *phase* and *consistency* (χ^2^(1) > 14; *p* < 0.001), interactions of the factor *phase* with both *direction* and *consistency* (χ^2^(1) > 5; *p* < 0.05), and a significant three‐way interaction direction*phase*consistency (χ^2^(1) = 5.82; *p* = 0.016). The post‐hoc test (Table 1) revealed that this pattern was due to two aspects: First, error sensitivity was larger in backward training than in the other conditions and, second, an effect of ITS consistency that occurred only in backward training. In that condition, error sensitivity was smaller (Wilcoxon signed rank test: *p* = 0.017) with inconsistent ITS (b_s = 0.025 [0.021]) than with consistent ITS.

**TABLE 1:** Median, interquartile range, and the *p*‐value of the paired comparisons of the error sensitivity $b_{s}$ in experiment 1 (*N* = 16)

Direction	Phase	Consis‐tency	*b_s_*: median [IQR]	Pairwise Wilcoxon signed rank test							
				Backward				Onward			
				Training		Washout		Training		Washout	
				Con.	Incon.	Con.	Incon.	Con.	Incon.	Con.	Incon.
Backward	Training	Con.	0.0337 [0.0303]	1.000	**0.017**	**<0.001**	**<0.001**	**0.049**	**<0.001**	**0.044**	**<0.001**
		Incon.	0.0251 [0.0209]	—	1.000	**0.001**	**0.003**	0.438	**0.006**	0.196	**0.001**
	Washout	Con.	0.0031 [0.0043]	—	—	1.000	0.234	**0.017**	**0.049**	0.352	0.642
		Incon.	0.0039 [0.0045]	—	—	—	1.000	0.070	0.196	0.408	0.501
Onward	Training	Con.	0.0086 [0.0238]	—	—	—	—	1.000	0.234	0.438	**0.013**
		Incon.	0.0071 [0.0064]	—	—	—	—	—	1.000	0.959	0.196
	Washout	Con.	0.0014 [0.0303]	—	—	—	—	—	—	1.000	0.569
		Incon.	0.0000 [0.0075]	—	—	—	—	—	—	—	1.000

Note

During backward training, $b_{s}$ was larger than during all other conditions. Error sensitivity was smaller with inconsistent than with consistent ITS but only during the backward training.

Significant p–values smaller than 0.05 are highlighted in bold.

The ART‐ANOVA applied to the fitted retention rate ($a_{s}$) showed significant main effects of the factors *direction* and *consistency* (χ^2^(1) > 7; *p* < 0.01) and no significant interaction effects. The factor phase did not show any significant main effect or interaction. The post‐hoc tests (Table 2) revealed that the retention rate was larger during the washout than during training. Only in the backward training condition was the retention rate larger (*p* = 0.023) with inconsistent than with consistent ITS.

**TABLE 2:** Median, interquartile range, and the p‐value of the paired comparisons of the retention rate $a_{s}$ in experiment 1 (*N* = 16)

Direction	Phase	Consistency	median [IQR]	Pairwise Wilcoxon signed rank test							
				Backward				Onward			
				Training		Washout		Training		Washout	
				Con.	Incon.	Con.	Incon.	Con.	Incon.	Con.	Incon.
Backward	Training	Con.	0.9909 [0.0091]	1.000	**0.023**	**<0.001**	**<0.001**	0.717	0.255	0.959	**0.020**
		Incon.	0.9953 [0.0029]	—	1.000	**<0.001**	**<0.001**	0.079	0.215	0.326	0.255
	Washout	Con.	0.9991 [0.0007]	—	—	1.000	0.877	**0.001**	**<0.001**	**0.010**	**0.003**
		Incon.	0.9990 [0.0015]	—	—	—	1.000	**0.002**	**0.002**	**0.007**	**0.017**
Onward	Training	Con.	0.9913 [0.0153]	—	—	—	—	1.000	0.379	0.438	**0.007**
		Incon.	0.9938 [0.0092]	—	—	—	—	—	1.000	0.918	**0.026**
	Washout	Con.	0.9967 [0.0176]	—	—	—	—	—	—	1.000	0.301
		Incon.	0.9971 [0.0022]	—	—	—	—	—	—	—	1.000

Note

During the washout, $a_{s}$ was larger than during training. Retention was larger with inconsistent than with consistent ITS but only during the backward training.

Significant p–values smaller than 0.05 are highlighted in bold.

Using Equation (3) and Equations ((9a), (9b), (9c)), the effect can also be expressed as an effect of ITS‐consistency on the initial adaptation speed which was 0.37 [0.34] %/trial in the condition with constant ITS and only 0.28 [0.23] %/trial with inconsistent ITS. This effect of the ITS‐consistency did not occur in the onward training or in the washout conditions.

**FIGURE 5:**
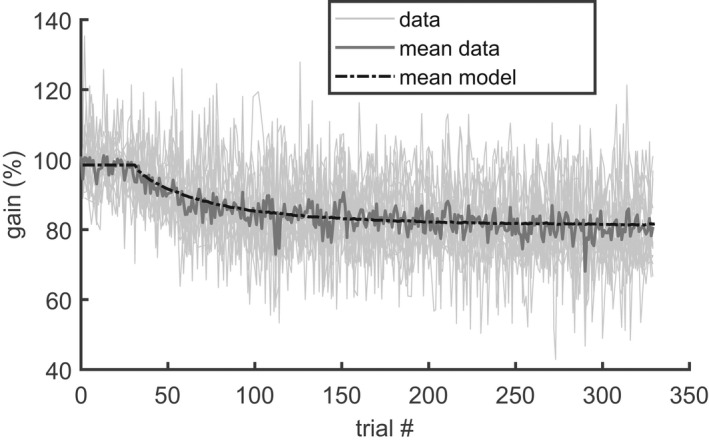
Light gray lines: saccade gain of all 16 subjects during the amplitude reduction with consistent ITS of 30% in “mixed training” (Exp. 1). Dark gray line: mean gain, averaged across all subjects. Dash‐dotted black line: mean model prediction, averaged across the four gain fields and all 16 subjects. The lack of systematic differences between the mean model and the mean data indicates that the applied one‐rate model captured the essence of the adaptation dynamics

Because our estimation procedure (Eggert et al., 2021) allowed estimation of the internal noise parameters ($\sigma^{2}$, $w_{\mathrm{ss}}^{2}$), the results also revealed that the variance of the execution noise, averaged across all adaptation conditions ($\sigma^{2}$=3.3e − 3 [2.8e − 3]) was much larger than the variance of the planning noise ($w_{\mathrm{ss}}^{2}$=9.4e − 6 [7.8e − 6]). With these variance parameters, the multi‐gainfield model predicted a residual gain variance of 5.2e − 3 [3.9e − 3] and a negative autocovariance (lag 1) of −3.0e − 4 [2.4e − 4].^1^ The observed auto‐covariance (lag 1) (−3.2e − 4 [4.7e − 4]) did not differ significantly (Wilcoxon signed rank test: *p* = 0.21) from this prediction. The observed residual gain variance (5.4e − 3 [3.9e − 3]) was slightly larger (Wilcoxon signed rank test: *p* = 0.02) than predicted. This may point either to components of the observed residual that are not captured by the noise model or even to systematic deviations between the observed and the predicted adaptation time course. However, comparing the mean time course of the modeled gain, averaged across the four gain fields and all subjects (Figure 5, dash‐dotted), with the mean observed gain, averaged across all subjects (Figure 5, dark gray solid) did not reveal systematic differences. This suggests that the introduction of a second internal memory state, as proposed in Ethier et al. (2008a), does not provide a substantial modeling advantage in our data. The lack of systematic differences shows that the small difference (0.2e − 3) between observed and predicted residual gain variance was due to small idiosyncratic gain changes that were not captured by the model. This does not suggest a major deficiency of the model because the autocovariance of the residual was correctly reproduced.

### 3.2 Single‐gainfield adaptation (experiment 2)

**FIGURE 6:**
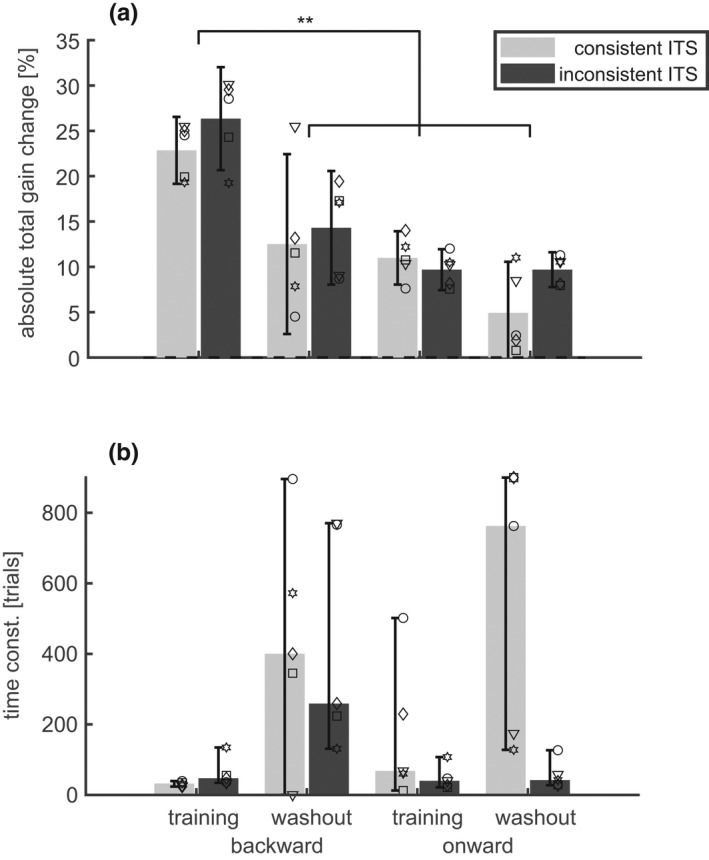
Adaptation dynamics in “single‐amplitude training” and subsequent washout (Exp. 2). The plots show the change (a: $\left|\Delta g_{2}\left(300\right)\right|$) of the adapted gainfield and its time constant (b: $\tau_{2}$) only for the adapted rightward saccades. All other legends correspond to those of Figure 4. Again, as in Exp. 1, neither the total adaptive gain change nor its time constant was affected by the consistency of the ITS

Figure 6a shows that the total gain change $\left|\Delta g_{2}\left(300\right)\right|$ in the “single‐amplitude training” depended on the different conditions in the same way as in the “mixed training” (Exp. 1). The adaptive change was larger for the backward/training condition (24.5 ± 3.7%) than for all the other conditions (10.35 ± 1.83%). The ANOVA on the absolute total gain change $\left|\Delta g_{2}\left(300\right)\right|$ showed the same significant effects of the factors *direction* (F(1, 4) = 39.1; *p* < 0.01), *phase* (F(1, 4) = 28.6; *p* < 0.01) and the interaction between both (F(1, 4) = 10.9; *p* < 0.05) as in Experiment 1. None of the main effects or interactions involving the factor *consistency* reached significance (*p* > 0.15).

The adaptation speed also showed a tendency of fastest adaptation during the backward training where the median [interquartile range] of the time constant $\tau_{2}$ was 38 [20] trials compared to 255 [177] trials in the other conditions. However, due to the large inter‐subject differences and the small number of subjects, neither the ART‐ANOVA nor the paired Wilcoxon signed‐rank test showed any significant effect of the factors *direction*, *phase*, or *consistency* on the time constant $\tau_{2}$ (Figure 6b).

### 3.3 Comparison of the absolute total gain change between training protocols

**FIGURE 7:**
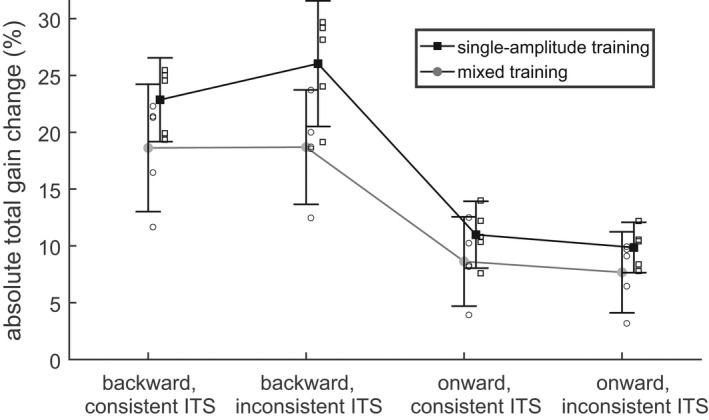
Comparison of the adaptive gain change between “mixed training” (gray circles: $\left|\Delta \bar{g}\left(300\right)\right|$) and “single‐amplitude training” (black squares: $\left|\Delta g_{2}\left(300\right)\right|$). Solid symbols: mean. Open symbols: individual data. Whiskers: 95% confidence interval of the mean. With the same number of training trials, adaptation was more efficient in single‐amplitude training than in mixed training

In the “single‐amplitude training” (Experiment 2), each saccade to the right contributed fully to the adaptation of the gain field for saccades with amplitudes of +16 deg. In contrast, in the “mixed training” (Experiment 1), the training of the 8 and 16 deg saccades to the right had to be shared between two different gain fields, each of which was trained with full efficiency not by 50% of all saccades, but only by 25%. Thus, qualitatively, the training in Experiment 2 is expected to be more efficient than the mixed training in Experiment 1. This expectation is confirmed by Figure 7 showing the comparison of the absolute total gain change during training between Experiment 1 (light gray diamonds: $\left|\Delta \bar{g}\left(300\right)\right|$) and Experiment 2 (black squares: $\left|\Delta g_{2}\left(300\right)\right|$). In the backward/training condition, the adaptive changes in the “mixed training” (18.7 ± 4.2%) were smaller than in the “single‐amplitude training” (24.5 ± 3.7%). An effect in the same direction but smaller in size occurred also in the onward/training condition (mixed: 8.2 ± 2.6%; single‐amplitude: 10.4 ± 0.9%). Statistically, this was confirmed by a repeated measures ANOVA on the absolute total gain change with the two factors *direction* (onward/backward) and *experiment* (1/2). Both main effects (*experiment*: F(1, 4) = 48.98; *p* < 0.01; *direction*: F(1, 4) = 41.47; *p* < 0.01) and the interaction (F(1, 4) = 13.51; *p* < 0.05) were significant.

**FIGURE 8:**
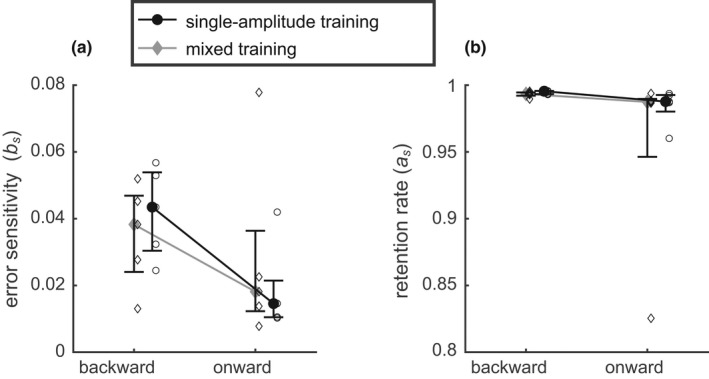
Comparison of error sensitivity (a) and retention rate (b) between “single‐amplitude training” (Exp. 2) and “mixed training” (Exp. 1). The solid symbols and the whiskers indicate median and quartiles. Open symbols show individual data averaged across the two conditions with consistent and inconsistent ITS. No significant differences were observed between the two training protocols

To test whether the multi‐gainfield model described the parallel training of 8 and 16 deg saccades correctly, the fitted model parameters were compared between the training phases of Experiments 1 and 2. Figure 8 shows that neither the retention rate ($a_{s}$=0.991 [0.017]) nor the fitted error sensitivity ($b_{s}$=0.031 ± 0.008) depended on the experiment. The repeated measures ART‐ANOVA on the retention rate $a_{s}$ with the factors *direction* (backward/onward) and *experiment* (1/2) revealed a significant main effect of the factor *direction* (χ^2^(1) = 6.82; *p* < 0.01) but no effect or interaction involving the factor experiment (*p* > 0.6). The error sensitivity $b_{s}$ was not significantly affected by either factor or their interaction (repeated measures ART‐ANOVA: χ^2^(1) < 2.5; *p* > 0.1).

The absence of any significant effect of the factor *experiment* on the model parameters $a_{s}$ and $b_{s}$ suggests that the differences of the adaptation between the training protocols (Figure 7) are sufficiently explained by the linear error weighting as implemented by the model of Tanaka et al. (2012).

### 3.4 Age dependency

**FIGURE 9:**
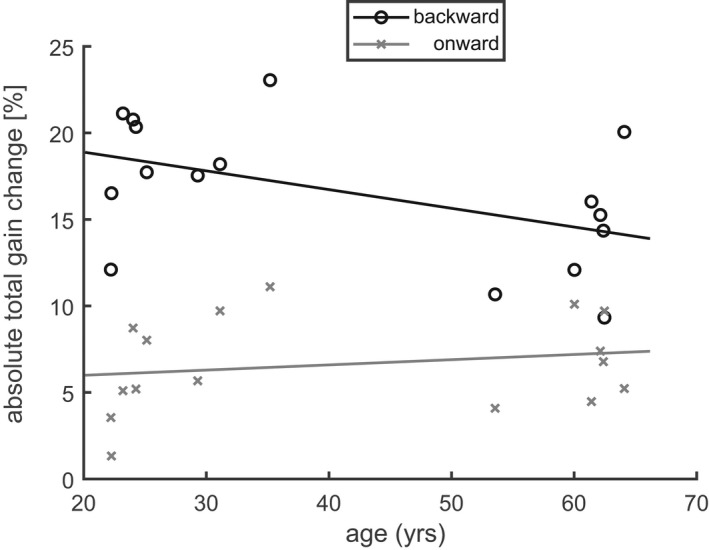
Age dependency of the absolute total gain change ($\left|\Delta \bar{g}\left(300\right)\right|$) during the training with onward (gray crosses) and backward (black circles) ITS in Experiment 1. Each symbol shows the mean of an individual subject across the two conditions with consistent and inconsistent ITS. Solid Lines: linear regression of $\left|\Delta \bar{g}\left(300\right)\right|$ as function of age. Backward adaptation (slope −0.11 ± 0.05%/year) but not onward adaptation (slope: 0.03 ± 0.46%/year) decreased with age

Figure 9 shows the dependency of the absolute total gain change $\left|\Delta \bar{g}\left(300\right)\right|$ during both backward (black) and onward (gray) training on age. The repeated measures model showed not only a significant main effect of the factor *direction* (F(1, 14) = 36.29; *p* < 0.001), indicating again larger changes in backward than in onward training. It also showed a significant interaction (F(1, 14) = 5.72; *p* < 0.05) between *direction* and *age*, indicating that the absolute gain change decreased with age in the backward training (slope of the linear regression: −0.11 ± 0.05%/year) significantly more strongly than in the onward training where the age dependency was effectively absent (slope: 0.03 ± 0.04%/year). Thus, the adaptive changes decreased with age in the backward training, but not in the onward training.

## 4 DISCUSSION

In summary, the results demonstrate that adaptation of saccades under mixed training conditions is well captured by the one‐rate model of Smith et al. (2006) in combination with a model extension for visuomotor adaptation of movements with multiple target positions (Tanaka et al., 2012), tailored to gain adaptation of saccades. This multi‐gainfield adaptation model is characterized by a linearly weighted contribution of the postsaccadic error of the current saccade in driving all adaptation of gain fields. Investigating the effects of direction of the ITS (onward/backward), the adaptation phase (training/washout), and the consistency of the ITS (consistent/inconsistent), we observed that the adaptive change was larger and occurred faster in the backward training than in all other conditions. The consistency of the ITS had no significant effect on the total adaptive gain change. The adaptation dynamics showed a larger error sensitivity and a smaller retention rate during backward training with consistent than with inconsistent ITS. For the onward training or any of the two washout conditions, no effects of ITS‐consistency were observed.

### 4.1 Robustness of total adaptive gain changes to error inconsistency

The current study did not find a significant effect of ITS‐consistency on the total adaptive gain change. This is consistent with the basic feature of linear adaptation models that the expected gain change depends only on the expected error signal but not on its variance. The control experiment (Experiment 2) showed that the observed robustness of the adaptive changes against differences in the ITS‐consistency was not specific to the mixed training protocol applied in Experiment 1 but manifest also with the single‐amplitude training protocol (open vs. solid bars in Figure 4, Figure 6) used by previous studies on this topic (Havermann & Lappe, 2010; Srimal et al., 2008).

This suggests that non‐linear effects of the error variance do not generally impair saccade adaptation. This conclusion is in line with the study by Srimal, Diedrichsen, Ryklin and Curtis (Srimal et al., 2008), in which they showed that the mechanism of saccade adaptation did not differ between a training with 2 deg backward ITS only and a training in which 2 deg backward and 1 deg onward ITS were presented in random order. Both the study of Srimal et al. (2008) and the current study have in common that the system was observed under closed‐loop conditions: The postsaccadic retinal error was controlled not directly but only indirectly by manipulations of the ITS. Therefore, the distribution of the postsaccadic error was not stationary. Its mean underwent a continuous decrease during the experiment due to the adaptive response of the subject.

In contrast, the study by Havermann and Lappe (2010), which found that the speed of the adaptive change decreased with increasing standard deviation of the postsaccadic error, was performed under open‐loop conditions, i.e., the postsaccadic visual error was directly controlled by the experimental setup and the time series of the error showed stationary statistics with constant mean and variance. Havermann and Lappe (2010) observed that under open‐loop conditions, adaptive changes proceed with nearly constant speed. Therefore, they used the average adaptation speed rather than the total adaptive change to quantify the effect of error variance. They observed for backward training with a postsacccadic retinal error with a standard deviation of 2 deg a decrease of the adaptation speed by about 26% with respect to a condition with constant postsaccadic retinal error. This finding is compatible with our observation that the error sensitivity and the initial adaptation speed were smaller with inconsistent ITS than with consistent ITS. Also the percent decrease of the initial adaptation speed (1 – 0.28/0.37 = 24%) in our Experiment 1 was similar to the effect (26%) reported by Havermann and Lappe (2010). The initial adaptation speed in our closed‐loop condition can be compared with the average adaptation speed during open‐loop condition because, at the beginning of the training, the adaptive response is still zero. Also, the standard deviation of the postsaccadic visual error (2.1 deg for 16 deg ITS) in the present experiment was similar to the one applied by Havermann and Lappe (2010). Nevertheless, in our data, the effect was restricted to the backward training and did not occur during the washout conditions or during onward training. It also did not affect the observed total adaptive changes in any of our closed‐loop training conditions. Thus, the effect of error consistency on adaptation speed was limited. This aspect of our data is not inconsistent with the inhibitory effect of error consistency on learning speed observed in previous studies (Havermann & Lappe, 2010). The absence of the effect in onward training and the washout conditions of the current study shows a lack of statistical support, which may be due to the smaller adaptive changes in these conditions and does not necessarily imply a lack of effect. However, the results indicate that under closed‐loop conditions, error consistency affects only the initial adaptation speed, but not the overall adaptation achieved after 300 trials.

This specificity of the effect is a characteristic feature of the linear adaptation model which predicts that the asymptotic gain change (Equation (4b)) decreases with decreasing $b_{s}$ and increases with increasing $a_{s}$. Therefore, the model is indeed very helpful to understand why both effects of error inconsistency on $b_{s}$ and $a_{s}$ compensate each other for the asymptotic gain change.

In contrast to the results of the current study, error variance was found to reduce the overall adaptive change in reaching movements (Albert et al., 2021). This may indicate a difference between the adaptive mechanisms of eye and arm movements.

### 4.2 The role of nonlinear mechanisms in saccade adaptation

Previous studies proposed a nonlinear adaptation mechanism in which the error sensitivity depend dynamically on the temporal history of error signals (Herzfeld et al., 2014). This mechanism, which can be characterized as a meta‐adaptation acting on error sensitivity, can explain the decrease of error sensitivity and initial adaptation speed we observed with inconsistent ITS in the backward training condition. The argument of the previous paragraph shows that the total asymptotic adaptive gain change depends not only on possible effects of error inconsistency on error sensitivity, but also on retention rate. Therefore, the model of Herzfeld et al. (2014), dealing only with the error sensitivity, would not be sufficient to predict adaptation speed or the total gain changes in adaptations with retention rates smaller than one ($a_{s}<1$).

Even though retention rates in our study were close to one (see Figure 8), it is important to note that in the mixed training paradigm, a given saccade amplitude is trained with the error signal at full efficiency only in a minority of trials. During the remaining trials, the adaptive state of any given saccade amplitude is diminished by repeated multiplication with the retention factor. This suggests that in mixed training, even retention factors close to one affect both adaptation speed and total gain change.

In a study on force‐field adaptation of arm movements (Huang & Shadmehr, 2009), it was found that retention rates measured during error‐clamp trials were larger after gradual than after abrupt training, and in visuomotor adaptation, retention rates tend to decrease after training with inconsistent error signals (Turnham et al., 2012). However, these results do not allow quantitative predictions about effects of error inconsistency on retention rates in saccade adaptation. Due to this uncertainty, the use of nonlinear meta‐adaptation models in closed‐loop saccade adaptation would be beneficial only if the parameters of these nonlinear models could efficiently be estimated. In the data of our experiment, this was not the case because we observed opposite effects of error inconsistency on error sensitivity and retention rate. According to Equation (4b), these two effects have opposing impacts on the overall adaptive gain change. Consequently, the total adaptive change provides only little information about the parameters of meta‐adaptation. Fitting models of meta‐adaptation from adaptation experiments like ours where the saccade gain asymptotically approaches a stationary value poses an ill‐conditioned problem. Vice versa, this consideration also explains why models including non‐linear effects on error sensitivity and/or retention rate are of limited value to explain the presented data.

The linear model makes the simplifying assumption that adaptation is driven by the postsaccadic retinal visual error. This seems to conflict with many previous studies (Bahcall & Kowler, 2000; Collins & Wallman, 2012; Havermann et al., 2014; Wong & Shelhamer, 2014) suggesting that saccade adaptation is not directly driven by retinal error but rather by the discrepancy between the postsaccadic target position and an internally generated prediction of that position. However, good performance of a model based on retinal error is expected when the predicted errors do not systematically differ from zero. Therefore, the good performance of the simple model in the current study is not an argument against the more detailed model, but only indicates that the conceptually important difference between retinal error and prediction error did not apply under the conditions of the current experiment.

### 4.3 Multi‐gainfield versus global adaptation

Rolfs et al. (2010) examined a mixed training condition that included not only targets on the horizontal meridian but also target positions in the entire frontal plane. This training involved, similar to the mixed training of the current study, parallel training of saccades belonging to different but overlapping gain fields. Rolfs et al. (2010) also compared this global training condition with a single‐amplitude training in which only saccades to the right were adapted. This condition differed from our single‐gainfield condition only in the size of the target steps (8 vs. 16 deg) and in the order of rightward and leftward saccades (random vs. strictly alternating). Rolfs et al. (2010) observed that the total adaptive change did not differ between the mixed training and the single‐amplitude training, a result which is incompatible with the predictions of the multi‐gainfield model. Therefore, Rolfs et al. (2010) suggested a global adaptation mechanism which acts at a larger spatial scale than the adaptation mechanisms bound to amplitude‐ and direction‐specific gain fields. This hypothesis states that single‐amplitude training triggers an amplitude‐ and direction‐specific adaptation mechanism, whereas mixed training triggers a global adaptation mechanism that is different from and more effective than parallel adaptation of multiple gain fields. An open question with respect to this hypothesis is which crucial factor determines which of the two mechanisms is predominant. In our experiment with horizontal saccades only, mixed training evoked smaller adaptive gain changes than the single‐amplitude training (Figure 7). This observation is, in contrast to the results of Rolfs et al. (2010), compatible with the predictions of the multi‐gainfield approach. Possibly, the broad distribution over the entire 2D space of the saccade vectors, in contrast to the restriction to the horizontal meridian in our experiment, is an important factor to elicit global adaptation. However, this conclusion is limited by the relatively small number of subjects that participated in our Experiment 2. This limitation is due to the fact that we designed Experiment 2 primarily as a control condition to test the effects of ITS consistency, rather than to examine the difference between global and local adaptation mechanisms.

### 4.4 Differences in saccade adaptation between backward and onward training

Since the early studies on saccade adaptation (Miller et al., 1981; Wolf et al., 1984), it is well known that the reduction of saccade amplitude induced by backward ITS proceeds faster and saturates at larger adaptive gain changes than the increase of saccade amplitudes induced by onward ITS with the same size. The generalization of saccade adaptation to saccades with different amplitudes and end positions also differs between onward and backward adaptation (Semmlow et al., 1989). Other studies focused on the specific effects of onward or backward adaptation on the velocity profile of the saccades (Collins et al., 2008; Ethier et al., 2008b; Frens & Van Opstal, 1997; Straube & Deubel, 1995). Even though the results of these studies are partially diverging (see Ethier et al., 2008b) for a discussion, all of them support the hypothesis that backward and onward adaptations rely on different mechanisms. Our observation that the error sensitivity was larger in the backward than in onward is consistent with these previous studies and indicates that the linearity of the adaptation model is violated.

The main new aspect of our experimental design is that it included not only onward and backward training but also the reverse adaptations during the washout conditions. The factorial analysis (Figure 4) showed that both the absolute total gain change and the time constant of the adaptation are subject to a significant interaction between the factors *direction* (onward/backward) and *phase* (training/washout). The most remarkable aspect of this interaction is that the gain decrease during the washout after the onward training was smaller and slower than the gain decrease during the backward training. This can partly be explained by the fact that the adaptive change achieved at the end of the onward training (7%; Figure 4a), and therefore the retinal error at the beginning of the following washout was much smaller than the retinal error at the beginning of the backward training (30%). However, this explanation is not sufficient because the same interaction effect of the factors *direction* and *phase* was also observed on the error sensitivity $b_{s}$ (rows 1 and 4 in Table 1). This suggests that the fast and efficient adaptation mechanism acting during backward training is specific not only to the direction of the required adaptive change as proposed by previous studies (Ethier et al., 2008b), but also to the sign of the actual saccade dysmetria. The same backward ITS was more efficient in reducing hypometric saccades than in reducing hypermetric saccades. There is of course the additional possibility that the adaptation mechanism is not only specific to the sign of the error and to the sign of the dysmetria but also to the history of the adaptive changes. However, such a mechanism of hyper‐learning alone cannot explain the finding of the current study that neither the total change nor the dynamics of the onward adaptation differed from the subsequent washout.

Further support for the hypothesis that backward saccade adaptation is achieved by a specific mechanism is provided by the observation that the total gain change decreased with increasing age in backward but not in onward saccade adaptation (Figure 9). However, the strength of this argument is limited by the fact that the number of participants was relatively small for an analysis of age dependence.

A dependence of error sensitivity and retention rate on error sign and adaptive state represents only one type of nonlinearity that can explain the differences between backward training and the other conditions. Recently, Masselink and Lappe (2021) presented a model explaining motor and perceptual adaptation through collective plasticity of spatial target percept, motor control, and visual prediction based on a corollary discharge (CD). In this model, the three corresponding gains (visual gain, motor gain, and CD gain) are adapted by a non‐linear learning rule that has three error sensitivities (one for each gain) as free parameters under the assumption of complete retention. Because of the different baseline values of the three gains, the model can also explain differences between onward and backward training.

### 4.5 Gain adaptation vs. amplitude adaptation

In a previous study (Ethier et al., 2008a) which also used the discrete‐time linear filter of Smith et al. (2006) in saccade adaptation, the internal states (x) represented the adaptive change of the saccade amplitude, and the input (u) driving the adaptation represented the ITS (expressed in deg). Therefore, this model can be considered a model of amplitude adaptation. In contrast, the current study used the same type of time‐discrete filter to model gain adaptation, in which the internal state x represents the gain change and u the normalized ITS (expressed as a fraction of the required saccade amplitude, Equation (1)). For the adaptation of only one saccade amplitude, gain adaptation and amplitude adaptation do not differ because of the linearity of the model. Dividing the input ($e\left(n\right)$) of a linear system by a constant value, leads, by definition, to the division of the output ($x\left(n\right)$) by the same value. However, the two models differ when multiple saccade amplitudes are trained in random order because the adaptation transfer depends strongly on whether it is expressed as a transfer of a gain change or as a transfer of an amplitude change. We choose the gain adaptation model because previous studies (Straube et al., 1997) showed an almost symmetric gain‐adaptation transfer between saccades with 5 and 15 deg amplitude: The gain‐adaptation transfer from trained 5 deg saccades to untrained 15 deg saccades (0.36) was not too different from the reverse transfer (0.28) from trained 15 deg saccades to 5 deg untrained saccades. Expressing this same transfer as a transfer of amplitude adaptation yields very asymmetric, namely 0.36·15/5 = 1.08 for adaptation transfer from 5 deg to 15 deg saccades and 0.28·5/15 = 0.09 for the reverse transfer. Thus, in terms of amplitude adaptation, the transfer from the small to the large saccades can be expected to be about ten times stronger than the transfer from large to small saccades. Consequently, in a model of amplitude adaptation, the transfer matrix C would be strongly asymmetric compared to the one to be used in gain adaptation (Equation (8)). With such asymmetry, and with the training protocol of Experiment 1, the solution of Equations (6)–(8) becomes a very unwieldy superposition of four exponential functions with different time constants and with different dynamic responses for each of the four adaptation fields. In contrast, our gain‐adaptation model predicts equal and parallel adaptation of all gain fields with a single exponential function (Equations ((9a), (9b), (9c))). The confirmation of this prediction (Figure 2) supports the gain‐adaptation model (Equations (6)–(8)).

The symmetry of the transfer matrix C about its diagonal mentioned in the previous paragraph concerns only the mutual transfer between two different saccade amplitudes. This symmetry must not be confused with the symmetry that refers to the transfer of the adaptation of a trained saccade of given amplitude to two other, untrained saccades. For example, many previous studies have shown that the adaptive transfer of a trained horizontal saccade to a larger untrained horizontal saccade is greater than that to a smaller one. (Collins et al., 2007; Noto et al., 1999). Such an asymmetry can of course be easily modeled with a symmetric transmission matrix C.

Further support for the multi‐gainfield adaptation model is provided by our control experiment because the same retention rates ($a_{s}$) and error sensitivities ($b_{s}$) were found for both protocols with single‐amplitude training and mixed training (Figure 8). This demonstrates that the same model could explain the adaptive changes in both experiments and confirms the assumption of linear superposition of adaptive changes in mixed training protocols (Tanaka et al., 2012). Previous studies already observed a linear superposition of the total adaptive changes in mixed training protocols (Semmlow et al., 1989). Moreover, the current study suggests that the linear superposition concerns not only the total adaptive change but also the dynamics of the adaptive process, and that the same gain transfer matrix C derived from a transfer after single‐amplitude training (Straube et al., 1997) is able to explain the transfer of gain adaptation in mixed training protocols.

At first glance, the assumption of a normalization of the retinal error and the state variable of the system may seem problematic. Why and how should the brain perform such a complicate algebraic operation as a division? However, this problem is a pseudo problem because it depends on the assumption that both the planned target amplitude and the postsaccadic retinal error are represented by the central nervous system within a linear metric. This is rather unlikely because it is known that motor maps such as the superior colliculus represented motor plans of saccade amplitude in a logarithmic distortion (Opstal & Goossens, 2008). It seems therefore reasonable to assume that adaptation, as far as it occurs on the level of motor planning, acts on a nonlinearly distorted representation with progressive space compression for larger amplitudes, and that sensory feedback of the saccade amplitude used to adapt such a motor representation is also transformed into the same metric. The Appendix shows that the adaptation of such a logarithmic motor map by means of a linear dynamic system can equivalently be expressed by a linear system acting on the normalized non‐logarithmic input. Thus, the normalization is needed only when input and output of the system are expressed in units of degree. In contrast, the adaptation of the logarithmic motor map realizes this normalization implicitly without need for an explicit division.

## 5 CONCLUSION

The current study provides evidence that multi‐gainfield saccade adaptation can be modeled successfully under the assumption that multiple amplitude‐specific planning circuits adapt independently driven by the weighted postsaccadic visual error. We applied and tested this model, previously developed in the context of adaptation of reaching movements (Tanaka et al., 2012), to saccade gain‐adaptation. The linearity of the model was challenged by a mixed training of saccades with different amplitudes, comparison of the adaptation between training and washout, and between different levels of error consistency. The model is supported by the observation that all tested conditions except the training with backward ITS, which showed especially large and fast adaptive responses, could be mimicked using similar model parameters. The observation that the dynamics of adaptive saccade amplitude reduction differs between backward training and washout after onward training suggests that the special role of the backward training, noticed by many previous studies, cannot be characterized as specificity to the direction of the adaptive change (or the direction of the visual error) alone but also includes specificity to the sign of the actual dysmetria of the saccade.

Error consistency did not affect the total adaptive changes in any of the conditions tested. In the healthy subjects who participated in the current study, doubling the standard deviation of the postsaccadic error from 1 deg to 2.1 deg did not impair the size of the adaptive changes. This suggests that the non‐linear inhibitory effects of error consistency on the adaptation speed (Havermann & Lappe, 2010; Herzfeld et al., 2014) do not play a major role in the asymptotic gain change. With respect to the original motivation of our research question, it seems therefore unlikely that increased saccade variability in cerebellar degenerative diseases would by itself explain the deficits of these patients in saccade adaptation.

## CONFLICT OF INTEREST

The authors declare that the research was conducted in the absence of any commercial or financial relationships that could be construed as a potential conflict of interest.

## Footnotes

^1^
Note that all variances and mean squares are given here as variance of the gain with respect to the saccade amplitude that guides the eye accurately onto the target and are therefore dimensionless. Thus, the observed mean squared residual of 5.4e − 3 corresponds to a standard deviation of the gain of $\sqrt{0.0054}=7.35\%$. For a saccade amplitude of 16 deg, this corresponds to a standard deviation of the saccade amplitude of 0.0735·16deg=1.18deg. The gain autocovariance of −3.2e − 4 corresponds for a 16 deg saccade to an amplitude autocovariance of $‐3.2e\;‐\;4\;∙16^{2}\;\deg^{2}=‐0.082\;\deg^{2}$.

## 1

A linear dynamic system of first order, applied to a logarithmic transformation of the planned saccade amplitude (A) is described by

(A1)
$$\frac{d}{\mathrm{dt}}\log\left(A\right)=‐\tilde{a}\left(log\left(A\right)‐log\left(A_{b}\right)\right)+\tilde{b}\left(log\left(A_{b}\right)‐\text{log}\left(A_{f}\right)\right)$$

where $A_{b}$ denotes the baseline amplitude, and $A_{f}$ the amplitude signaled by the sensory feedback. For $0<\tilde{a}<1$, the homogeneous and stationary solution of this differential equation is $A=A_{b}$. The driving input $log\left(A_{b}\right)‐\text{log}\left(A_{f}\right)$ represents the difference between the expected and the actual feedback, expressed in the logarithmic metric of the planned amplitude. Using the linearization $\text{log}\left(A\right)=log\left(A_{b}+z\right)\approx \text{log}\left(A_{b}\right)+z/A_{b}$ yields

(A2)
$$\frac{d}{\mathrm{dt}}\log\left(A\right)\approx \frac{\dot{z}}{A_{b}}\approx ‐\tilde{a}\frac{z}{A_{b}}+\tilde{b}\frac{A_{b}‐A_{f}}{A_{b}}$$

where $z=A‐A_{b}$ denotes the adaptive change of the planned amplitude. If the feedback $A_{f}$ is systematically distorted by the ITS ($A_{f}=A‐ITS)$, we obtain

(A3)
$$\frac{\dot{z}}{A_{b}}\approx ‐\tilde{a}\frac{z}{A_{b}}+\tilde{b}\frac{ITS‐z}{A_{b}}=‐\left(\tilde{a}+\tilde{b}\right)\frac{z}{A_{b}}+\tilde{b}\frac{\mathrm{ITS}}{A_{b}}$$

which is the time‐continuous form of

(A4)
$$\frac{z\left(n+1\right)}{A_{b}}=\left(a_{s}‐b_{s}\right)\frac{z\left(n\right)}{A_{b}}+b\frac{\mathrm{ITS}}{A_{b}}$$

with $a_{s}=1‐\Delta t\tilde{a}$ and $b_{s}=\Delta t\tilde{b}$. The time‐discrete gain‐adaptation model (Equation (A4)) is identical to the amplitude‐adaptation model of (Smith et al., 2006) after normalization of the input (ITS) and the output (z) to the baseline saccade amplitude ($A_{b}$). The above consideration shows that a linear adaptation dynamic applied to a logarithmic metric (Equation (A1)) includes this normalization implicitly without the need for an explicit internal representation of the gain or of the gain change. Even though this normalization has no effect on the solution of Equation (A4) for $z\left(n\right)$ when $A_{b}$ is constant, the normalization becomes relevant when multiple saccade amplitudes are adapted in parallel.

The normalization in Equation (A4) is performed with respect to the baseline amplitude $A_{b}$, whereas the model of the current study (Equations (1) and (2)) normalizes with respect to the ideal amplitude R required to hit the target accurately. This difference is of minor importance because the actual values of the saccade gain are only slightly smaller than one.
